# A comparative analysis of game‐based learning and conventional learning in dental education

**DOI:** 10.1002/jdd.13747

**Published:** 2024-10-22

**Authors:** Fernando Fernández‐Gómez, Maria Cosin‐Villanueva, Pedro Almiñana‐Pastor, Andrés López‐Roldán

**Affiliations:** ^1^ Department of Stomatology, School of Medicine and Dentistry Universitat de Valencia Valencia Valencia Spain

**Keywords:** dental education, educational technology, periodontics, preclinical education, serious games

## Abstract

**Objectives:**

To evaluate the efficacy of traditional teaching versus traditional teaching supplemented by serious gaming in imparting knowledge of periodontal indices among dentistry students. Additionally, the study seeks to measure the level of satisfaction among students engaging with the assessed teaching method.

**Materials and method:**

This comparative study was approved by the ethics committee of the University of Valencia with file number: 2479311. A sample of 61 subjects was divided randomly into two groups: the test group (*n* = 36) and the control group (*n* = 25). Baseline knowledge of community index of periodontal treatment need was assessed through a questionnaire completed by both groups before receiving a comprehensive explanation of the topic. The test group, in addition to the conventional explanation, received supplementary training via an educational gaming experience. Both groups underwent a final evaluation and, subsequently, a satisfaction survey was completed by the test group. Descriptive and inferential analyses were performed using a non‐parametric Brunner–Langer model. The relationship between scores was examined using Mann–Whitney and Wilcoxon tests, with a significance level set at α = 0.05.

**Results:**

The training, in general, was effective as both the test group (*p* = 0.003) and the control group (*p* = 0.015) demonstrated an increase in scores with both teaching modalities, but no significant difference was observed between the groups. The test group expressed a high level of satisfaction with the instructional approach.

**Conclusion:**

No statistically significant difference in learning outcomes was identified between the traditional lecture teaching method and the approach supplemented by gaming. There was an elevated level of student satisfaction with the gaming method.

## 1 INTRODUCTION

Before students start working on real patients, it is crucial for them to practice and gain competences in the field.^1^ This process can be facilitated by the advancement toward an era of “digital dentistry.”^2^

Serious gaming allows students to acquire knowledge interactively and outside of the classroom environment. The current generation of students, Generation Z, is experiencing a noticeable shift from conventional learning methods toward exploratory and digital learning.^3^, ^4^ Games incorporated into educational activities can take the form of modified commercial games or serious games, in a computerized or analog format.^5^, ^6^

Immersify Education (Manchester, UK) offers an educational platform designed as a virtual simulator for learning theoretical concepts. Compatible with various operating systems, this platform covers a diverse range of topics, including anatomy, dental prosthetics, periodontology, oral surgery, oral medicine, cariology, radiology, and restorative dentistry. Users can access content through the application or interact with a physical study model via a camera.^7^

In the Periodontology I section, Immersify Education allows access to a series of tools to study concepts related to periodontics. The “Perio Screening” feature is a learning game that simulates a periodontal examination in the mouth of a hypothetical patient. Up to three mistakes are allowed per game, rewarding correct answers with “medals,” and penalizing incorrect ones with the deduction of “lives.” The objective of the game is to correctly identify the community index of periodontal treatment needs (CPITN) values on the probed teeth. As one correctly identifies CPITN codes, medals are rewarded and new scenarios are “unlocked.” Like many other mobile application games, the end goal is to advance in the game, “unblocking” as many “levels” as possible, and thus gaining the highest amount of “medals” without losing all 3 “lives.”^7^

The world is evolving at an astronomical pace, and students are too. Given the limited existing information in the current literature, the primary objective of this study is to evaluate the efficacy of traditional teaching versus traditional teaching supplemented by serious gaming in imparting knowledge of periodontal indices among dentistry students, through an evaluation questionnaire. The secondary objective is to measure the level of satisfaction among dentistry students who use serious games as a complementary method to traditional teaching.

## 2 MATERIALS AND METHOD

This comparative study was approved by the ethics committee of the University of Valencia with file number: 2479311.

The gaming platform used in this investigation was the Immersify Education: Perio Screening mobile application, and the research was conducted at the Faculty of Medicine and Dentistry of the University of Valencia.

The research sample consisted of 61 4th and 5th‐year dentistry degree students from the University of Valencia, randomly assigned to two groups:

- Control group: received only conventional education.
- Test group: received conventional education as well as the gaming experience.

The students were invited to participate voluntarily in the study and were selected consecutively as they accepted through the signing of the informed consent. To be included in the study, the students had to be enrolled or have completed the Periodontology course. The exclusion criteria were not applied.

**FIGURE 1:**
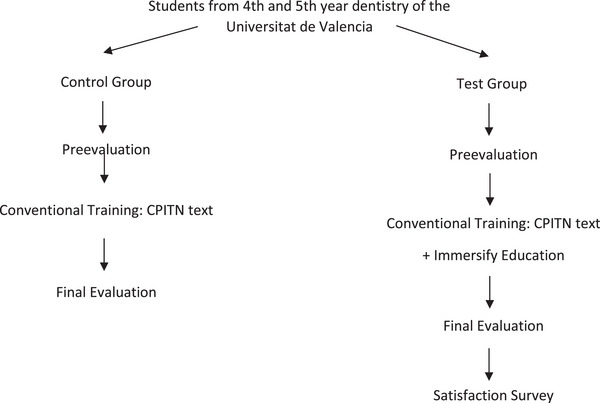
Methodology of the study.

An evaluation of the learning was conducted by adapting the method used to assess teaching through serious gaming by Amer et al.^3^ and Tubelo et al.^8^ The students followed the protocol described in Figure 1.

The preevaluation and final evaluation questionnaires were identical, and consisted of 10 multiple choice questions, each with four possible answers. The questionnaires were divided into two sections, based on the aspect that each one evaluated. The first part consisted of five theoretical questions about the CPITN and its interpretation, primarily including questions about how the CPITN index is used and the meanings of the different values. The second part consisted of clinical cases for the student to diagnose, providing information about a hypothetical patient and requiring the student to assign a CPITN value in each case. Each correct answer was worth two points to obtain a maximum score of 10 points in each section. It was not compulsory to answer every question, with the questions not answered or answered incorrectly having received a score of zero.

The conventional explanation of the CPITN was done using an excerpt from the widely referenced book *Clinical Periodontology and Implant Dentistry* by Jan Lindhe et al.^9^ commonly used in dentistry faculties. Each student was allowed 15 min for reading and the experimental group was allowed an additional ten minutes to engage with the gaming experience.

In addition, the experimental group was then required to complete a survey of satisfaction to evaluate their perception of the gaming experience. For the development of this survey, we based our approach on the previously published studies by Buajeeb^10^ and Arayapisit^11^ which evaluated the perceived ease of use, usefulness and enjoyment as the fundamental determinants of user acceptance in information technology as described by Davis.^12^ The satisfaction survey was comprised of five questions and employed a four‐point Linkert scale for participants to express their degree of satisfaction with the supplementary use of gaming in dental education.

### 2.1 Statistical methodology

The evaluation questionnaires provided theoretical and practical scores, each measured on a scale of 0–10. A total score, representing the average of both components, was also calculated. These variables were the primary metrics for the study.

A Shapiro–Wilk test concluded that the sample clearly followed asymmetric distributions. Therefore, the analysis was nonparametric.

A descriptive analysis and an inferential analysis were carried out, for which a non‐parametric Brunner–Langer model for longitudinal data was estimated. To explore the relationship between the grades obtained and the teaching method implemented, the Bonferroni correction was applied.

The Mann–Whitney test was implemented to compare the grades between the two groups, while the Wilcoxon test was utilized to assess whether there was a significant change in grade within each group over time.

The chosen level of significance for the analyses was set at 5% (α = 0.05), allowing for the identification of meaningful differences in the test scores obtained.

## 3 RESULTS

The sample consisted of 61 students was divided randomly into two groups: the test group (*n* = 36) and the control group (*n* = 25). The sample was comprised of 61 students from 4th and 5th year enrolled in the Dentistry degree. They were born either in the year 2000 or 2001. The test group consisted of 14 male students (38.9%) and 22 female students (61.1%). The control group consisted of 9 male students (36%) and 16 female students (64%).

### 3.1 Questionnaires

Tables 1 and 2 describe the grades achieved in the pre‐evaluation and in the final evaluation for each group, respectively.

**TABLE 1:** Pre‐evaluation scores (T0) according to group (scale 0‐10).

		Total theory T0		Total practical T0		Total score T0	
	*N*	Mean	Standard deviation	Mean	Standard deviation	Mean	Standard deviation
**Test**	36	9.1	1.2	7.6	1.8	8.3	1.2
**Control**	25	9.1	1.3	7.4	2.6	8.2	1.4
**Total**	61	9.1	1.2	7.5	2.1	8.3	1.3

**TABLE 2:** Final evaluation scores (T1) according to group (scale 0–10).

		Total theory T1		Total practical T1		Total score T1	
	*N*	Mean	Standard deviation	Mean	Standard deviation	Mean	Standard deviation
**Test**	36	9.4	0.9	8.7	1.7	9.1	1.1
**Control**	25	9.7	1.2	8.1	2.5	8.9	1.5
**Total**	61	9.5	1.1	8.5	2	9	1.3

Table 3 presents the difference in the scores of the pre‐evaluation and the final evaluation of each group.

**TABLE 3:** Difference T1‐T0 scores according to group.

		**Difference theory**		**Difference practical**		**Difference total score**	
	***N***	**Mean**	**Standard deviation**	**Mean**	**Standard deviation**	**Mean**	**Standard deviation**
**Test**	36	0.39	1.64	0.95	2.26	0.75	1.59
**Control**	25	0.56	1.69	0.72	1.99	0.64	1.32
**Total**	61	0.46	1.65	0.95	2.15	0.70	1.48

### 3.2 Intragroup results

An ANOVA‐type statistical test (ATS test) of the Brunner–Langer model revealed that, in terms of theoretical knowledge, the training was effective overall (time effect *p* = 0.009) for both groups (*p* = 0.432).

Bonferroni tests further indicated that:

- In the conventional training group, there was a significant increase in the theoretical score from the pre‐evaluation to the final evaluation (*p* = 0.026).
- In the gaming group, the increase in the theoretical score did not reach statistical significance (*p* = 0.410).

The ATS test of the Brunner–Langer model revealed that, in terms of practical knowledge, the training was effective overall (time effect *p* < 0.001) for both groups (*p* = 0.349).

Bonferroni tests further indicated that:

- In the conventional training group, there was a relevant increase in the practical score without reaching statistical significance (*p* = 0.063).
- In the gaming group, there was a significant increase in the practical score from the pre‐evaluation to the final evaluation (*p* = 0.003).

The ATS test of the Brunner–Langer model revealed that, overall, the training was effective (time effect *p* < 0.001) for both groups (*p* = 0.786).

Bonferroni tests further indicated that:

- In the conventional training group, there was a significant increase in the overall grade from the pre‐evaluation to the final evaluation (*p* = 0.015).
- In the gaming group, there was also a statistically significant increase in the overall grade (*p* = 0.003).

### 3.3 Intergroup results

The ATS test results for the theoretical evaluation indicated that the test scores of both groups were similar in both the pre‐evaluation (*p* = 0.164) and the final evaluation (*p* = 0.432).

Bonferroni tests affirmed that there were no significant differences in the grades of both groups during both the pre‐evaluation (*p* = 1.000) and the final evaluation (*p* = 0.148).

The ATS test results for the practical evaluation indicated that the test scores of both groups were similar in both the pre‐evaluation (*p* = 0.661) and the final evaluation (*p* = 0.349).

Bonferroni tests affirmed that there were no significant differences in the grades of both groups during both the pre‐evaluation (*p* = 1.000) and the final evaluation (*p* = 0.1000).

The ATS test results for the overall evaluation indicated that the test scores of both groups were similar in both the pre‐evaluation (*p* = 0.821) and the final evaluation (*p* = 0.786).

Bonferroni tests affirmed that there were no significant differences in the grades of both groups during both the pre‐evaluation (*p* = 1.000) and the final evaluation (*p* = 0.1000).

### 3.4 Satisfaction survey

The internal consistency of the questionnaire was measured using Cronbach's Alpha, which showed a value of 0.664, indicating a questionable internal consistency.

**FIGURE 2:**
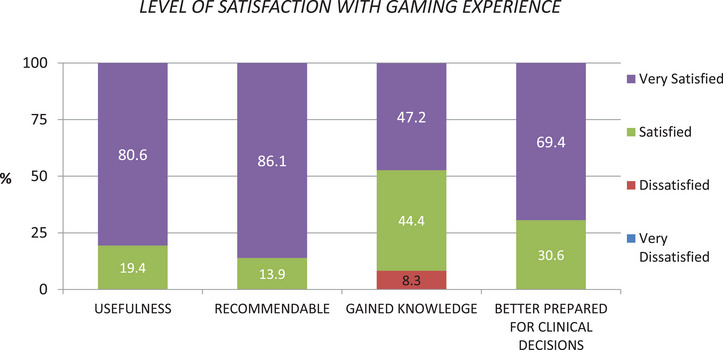
Level of satisfaction with gaming experience.

The results of the satisfaction survey are represented graphically in Figure 2. Most of the students claimed to be satisfied or very satisfied with the gaming experience and in general they felt better prepared for clinical practice with the use of the CPITN periodontal index.

## 4 DISCUSSION

In the context of comparing learning methods in dentistry, it is important to acknowledge the limitations of this investigation. This study serves as a pilot, providing a foundation for future investigations into teaching methods, particularly in the field of periodontics and the learning of periodontal indices. It would also be interesting to conduct more research in the future, with a larger sample size and including different variables, to assess what type of results can be achieved with alternative didactic methods.

Both groups, using different learning methods, improved their test scores in a similar way. Therefore, we can conclude that both methods are equally effective in teaching dental students the CPITN index.

A significant increase in the theoretical test score was observed in the traditional training group, while the group that received education complemented by gaming did not manage to improve the score significantly. It is plausible that this outcome was influenced by the students’ ability to better retain information in the short term when provided with the data immediately before the assessment. It would be interesting to assess long‐term knowledge retention in future studies and compare the results of both groups.

The analysis of practical test scores revealed a notable increase in both groups, with the gaming group reaching statistical significance. This could be due to the gaming group having engaged more in practical rehearsal of the CPITN index, leading to greater proficiency with its application.

Comparing one teaching method with the other, no statistically significant differences were observed in either the theoretical or practical test scores. This suggests that both methods were equally effective in facilitating student learning of the CPITN index. Increasing the sample size and refining the methodology could obtain significant results in these comparisons thus making it interesting to continue researching the effect of these new educational methods. The degree of student satisfaction with both methods was analyzed, and the results showed that students were highly satisfied with the gaming method, which reinforces the idea that, in new digital times, teaching should adapt and take into consideration the new tools at our disposal.

Similar studies in existing literature have reported results consistent with these findings. Specifically, new methodologies, including serious gaming, have not shown a significant increase in theoretical knowledge compared to traditional methods,^3^, ^5^, ^6^, ^8^, ^13^ however, they have consistently obtained high levels of satisfaction from both students and teachers.^3^, ^5^, ^6^, ^8^, ^13^ This suggests that, while not necessarily boosting theoretical understanding significantly, these modern methodologies are more aligned with the preferences of contemporary learners. In different fields of medicine and dentistry, several authors have found similar results. Bartolomé et al.^14^ also found similar results regarding the self‐perception of the students, who felt better prepared and with a greater sense of clinical competence. Some authors even suggest that, on a practical level, students are better prepared after utilizing some type of simulation.^15^

Although the results are similar to those of conventional training, more research and development are needed in order to implement this type of methodology regularly and absolutely in the health sciences.^16^, ^17^, ^18^

In the context of comparing learning methods in dentistry, it is important to acknowledge the limitations of this investigation. This study serves as a pilot, providing a foundation for future investigations into teaching methods, particularly in the field of periodontics and the learning of periodontal indices. It would also be interesting to conduct more research in the future, with a larger sample size and including different variables, to assess what type of results can be achieved with alternative didactic methods. Another limitation is the lack of consideration of demographic parameters. For future investigations, it could be interesting to take into extensive account sample demographics and not consider the sample totally homogenous.

## 5 CONCLUSION

Both teaching methods proved effective in facilitating student learning of the CPITN index, with no significant differences indicating superiority of one method over the other.

The students who used the game expressed a high degree of satisfaction with the new teaching tool and stated that they felt better prepared than with the traditional method for using the CPITN index. The results of this study point out that the teaching methodology should evolve and adapt to new generations of students. Considering that they can obtain the same or better results, students are more satisfied using these new didactic methods.

## CONFLICT OF INTEREST STATEMENT

The authors declare no conflicts of interest that could be perceived as prejudicing the impartiality of the research reported.
